# Simulation-Oriented Rule-Driven Geometric Modeling of Polymer-Fiber Weft-Knitted Structures for Moisture-Transfer Prediction

**DOI:** 10.3390/polym18162026

**Published:** 2026-08-21

**Authors:** Miao Miao, Nana Li, Hao Zhang, Yuxiao Tang, Tianqi Yang, Xiaodong Zhang

**Affiliations:** 1 School of Textile Science and Engineering, Tiangong University, No. 399 Binshui Xi Road, Xiqing District, Tianjin 300387, China; 2020010040@tiangong.edu.cn (M.M.); 2210110029@tiangong.edu.cn (H.Z.); tangyuxiao@tiangong.edu.cn (Y.T.); 2520010050@tiangong.edu.cn (T.Y.); 2 Cangzhou Institute of Tiangong University, Cangzhou 061000, China; 3 State Key Laboratory of Precision Measuring Technology and Instruments, Laboratory of Micro/Nano Manufacturing Technology, Tianjin University, Tianjin 300072, China; zhangxd@tju.edu.cn

**Keywords:** polymer-fiber textiles, weft-knitted structures, rule-driven geometric modeling, structure–property modeling, meshable simulation input, unidirectional moisture transfer

## Abstract

Polymer-fiber weft-knitted textiles are widely used in functional apparel and moisture-management materials, but their complex loop topology, yarn-level porosity, and interlayer hierarchy make simulation-oriented geometric modeling challenging. Conventional control-point and interpolated-curve methods often have limitations in representing knitting actions, maintaining yarn-path continuity, and generating meshable geometries. This study proposes a rule-driven geometric modeling method for polymer-fiber weft-knitted structures using the yarn centerline as the basic geometric carrier. Knitting actions, including knit, tuck, float, plating, and double-needle-bed assignment, are converted into reusable local path-generation rules and integrated through pattern-matrix input, action recognition, parametric centerline generation, continuous stitching, and standardized output. The method represents single-bed and double-bed structures within a unified framework, including plain, jacquard, plated, tuck, rib, interlock, half-cardigan, full-cardigan, and purl structures. Compared with an interpolated-curve method, the curvature-jump rate of four representative structures decreases from 40.78–69.23% to 0–0.31%, with markedly reduced maximum bending angles. Mesh-generation results show continuous meshes with improved element quality for complex double-bed structures. A moisture-transfer simulation of a fully plated plain-knitted structure gives one-way transport indices of −122.3187 and 122.2472 for face- and back-side liquid entry, with relative errors of 1.74% and 0.50% compared with experiments. These results indicate that the proposed method provides reproducible and meshable geometric input for structure–property modeling and moisture-transfer prediction of polymer-fiber knitted textiles.

## 1. Introduction

Polymer-fiber weft-knitted textiles are an important class of flexible porous materials for functional apparel, moisture-management fabrics, and wearable textile systems. They are formed by the intermeshing of continuous yarns through knitting actions such as knit, tuck, and float, and therefore exhibit pronounced multilevel characteristics, strong topological constraints, and high designability [1,2]. Unlike woven fabrics, which feature relatively regular warp–weft interlacing structures [3], loop units in weft-knitted structures present more-complex spatial relationships involving bending, turning, interlayer nesting, and local yarn overlap [4,5]. This hierarchical three-dimensional architecture determines not only the appearance, thickness, and pore distribution of the fabric but also the liquid transport pathways and geometric requirements for numerical analysis [6,7]. Therefore, establishing a reproducible three-dimensional model that can faithfully characterize yarn-level topology and geometric features is essential for structural analysis, structure–property prediction, and simulation-assisted design of polymeric knitted textiles.

From the perspective of polymer science, knitted fabrics can be regarded as hierarchical polymer-fiber assemblies in which material wettability, yarn-scale porosity, loop topology, and fabric-level channels jointly determine macroscopic transport behavior. Unlike conventional bulk polymer materials, the functional performance of knitted textiles is strongly influenced by their multiscale structural organization [8]. Consequently, an effective geometric representation should reproduce yarn morphology while maintaining continuous and meshable structures suitable for subsequent computational analysis [9].

In recent years, extensive research has been conducted on hybrid composites and natural fiber-reinforced polymer materials. These studies have mainly focused on material-level optimization, including fiber hybridization strategies, interfacial modification, mechanical reinforcement, and functional-property enhancement [10]. Such investigations have significantly advanced the understanding of polymer composite design and performance improvement. However, most hybrid composite studies consider reinforcement morphology as a predefined material component and primarily concentrate on composition–property relationships rather than the digital reconstruction of complex textile architectures. For knitted textile systems, the primary challenge is therefore not only material optimization but also the conversion of hierarchical yarn arrangements and topological structures into reliable computational representations [11].

To clarify the differences between previous polymer composite studies and the present investigation, representative studies related to polymer composites, fiber-reinforced simulations, and knitted textile modeling are summarized in Table 1. As shown in Table 1, previous studies have mainly addressed material reinforcement mechanisms, constitutive modeling, or performance prediction based on predefined geometrical configurations [12,13,14,15]. Although these approaches have greatly contributed to polymer material development, they generally lack automated approaches for generating continuous yarn-level geometries of complex knitted architectures. In contrast, the present work focuses on establishing a rule-driven geometric modeling framework that directly transforms knitting structural information into continuous and meshable three-dimensional models, thereby addressing the gap between knitted textile design and numerical simulation.

Existing studies on knitted textile geometric modeling mainly focus on representing yarn trajectories and reconstructing three-dimensional loop architectures. Among these approaches, Peirce-loop-based models have provided the theoretical foundation for knitted fabric geometry representation [16]. Peirce [17] established the classical geometrical principles of functional fabric design by describing the spatial arrangement of yarn loops, while Lesf and related analytical loop models [18] further developed mathematical descriptions of plain-knitted loops based on idealized loop shapes. These models successfully captured the basic geometric characteristics of single jersey structures and provided important theoretical references for subsequent knitted fabric simulations. However, because these models were mainly derived from ideal loop assumptions, their extension to complex knitting operations involving tuck, float, plating, and multi-layer structures remains limited.

To overcome the limitations of idealized loop descriptions, subsequent studies introduced analytical and computational geometric models to improve the flexibility of knitted textile representation. Cheng et al. [19] developed a matrix-transformation-based modeling method for partial knitted fabrics, while Yekrang and Semnani [20] established a rheological model for tubular weft-knitted textiles. Kurbak and Soydan [21] proposed a geometrical model for 1 × 1 purl fabrics, and Liu and Long [22] investigated three-dimensional simulation of plain weft-knitted fabrics through yarn-path reconstruction. These studies expanded the mathematical description of knitted structures; however, most models were developed for specific architectures and relied on predefined geometric relationships, limiting their extension to diverse knitting actions and complex structures.

Beyond analytical models, several studies have explored automatic yarn-level geometry generation for numerical simulation. Yuksel et al. [23] investigated yarn-level knitted cloth simulation based on prescribed knitting structures. These studies demonstrated the feasibility of rule-based geometric generation, but their modeling strategies were generally coupled with specific structural assumptions and lacked a unified framework for different knitting operations. More recently, yarn-level and multiscale simulation approaches have further improved the geometric accuracy and simulation capability of knitted textile models. Wadekar et al. [24,25] developed optimized yarn-level geometries for finite-element analysis of weft-knitted fabrics, Kaldor et al. [26] established a computational yarn-level knitted cloth simulation method, and Oliver et al. [27] proposed a nonlinear multiscale modeling framework for three-dimensional knitted textiles. Ru et al. [5] further demonstrated yarn-level modeling for fancy weft-knitted fabrics. Although these approaches significantly improved geometric fidelity and simulation performance, they mainly focused on specific fabric structures or accurate reconstruction of individual models. Therefore, a unified framework that simultaneously integrates knitting-action abstraction, parametric geometry generation, structural extensibility, and simulation-oriented meshability remains insufficiently developed [28].

Based on the above analysis, the remaining challenge is not merely the representation of individual knitted structures, but the establishment of a unified computational framework that can automatically transform knitting information into simulation-ready three-dimensional geometries. Therefore, this paper proposes a rule-driven three-dimensional geometric modeling method for polymer-fiber weft-knitted structures oriented to meshable numerical-simulation input. Rather than developing new composite materials, this work addresses the digital representation of hierarchical knitted architectures. The proposed method uses the yarn centerline as the fundamental geometric carrier and transforms knitting actions, including knit, tuck, float, plating, and double-needle-bed assignment, into reusable local path-generation rules. Through pattern-matrix representation, action recognition, parametric centerline generation, continuous stitching, and standardized geometric output, the proposed framework enables automated construction of complex single-bed and double-bed knitted structures.

Although moisture-transfer prediction is demonstrated using a representative fully plated knitted structure, the primary objective of this study is to validate the universality of the proposed geometric modeling framework rather than to establish a material-specific moisture-transfer database. Therefore, the applicability of the proposed method is further evaluated from multiple structural perspectives, including different knitted architectures, geometric stability, mesh-generation adaptability, and simulation compatibility. The main contributions of this study are summarized as follows:

(1) A rule-driven geometric modeling framework is established to convert knitting actions into executable geometric generation rules, enabling automated transformation from structural descriptions to three-dimensional yarn geometries.
(2) A unified parametric centerline representation is developed for polymer-fiber knitted structures, allowing various structures including plain, jacquard, plated, tuck, rib, interlock, cardigan, and purl fabrics to be described within the same modeling framework.
(3) A comprehensive evaluation strategy is introduced by analyzing centerline geometric stability, mesh-generation adaptability, and moisture-transfer prediction capability, demonstrating that the generated geometries can provide reliable input for subsequent multiphysics simulation and structure–property analysis.

Through these developments, this study provides an extensible and reproducible geometric modeling approach for complex polymer-fiber knitted textiles and establishes a computational foundation for simulation-assisted functional fabric design.

## 2. Materials and Methods

For clarity, the major symbols, parameters, and abbreviations used throughout this manuscript are summarized in Table 2.

The investigated system is a polymer-fiber hierarchical porous textile composed of continuous polymer yarns. In this study, the experimental validation fabric was manufactured using plated polymer yarns, including a face yarn of 75D/144F and a ground yarn of 75D/12F. Here, D represents yarn linear density (denier), and F represents the number of filaments. The polymer composition and fiber morphology determine the intrinsic material-related properties, including surface wettability, yarn permeability, and liquid–fiber interaction characteristics, which influence moisture transport behavior together with fabric-scale geometric structures. In the present computational framework, the geometric modeling process is independent of a specific polymer type. Instead, polymer-dependent parameters are introduced during subsequent multiphysics simulation. Therefore, different polymer-fiber systems can be investigated by updating material properties while maintaining the same geometric-generation rules. This separation between structural representation and material parameters enables the proposed method to be extended to various polymeric textile systems.

### 2.1. Overview and Algorithm of the Rule-Driven Geometric Modeling Method

The proposed approach is a general rule-driven geometric modeling method rather than a software-specific modeling procedure. The core objective is to transform knitting structural information into three-dimensional simulation-oriented yarn geometries through a unified computational framework. The proposed method consists of three fundamental components: (1) parametric yarn-centerline representation, (2) knitting-action-based geometric transformation rules, and (3) topology-preserving assembly strategy.

To generate three-dimensional geometric models of polymer-fiber weft-knitted structures that can be directly used in subsequent numerical simulations, a centerline geometric modeling method based on knitting rules is established. This method uses the yarn centerline as the fundamental geometric carrier and transforms structural information from manually defined control-point descriptions into an automated modeling process consisting of pattern matrix, action recognition, local rules, continuous stitching, and standardized output. The modeling objective is not to fully reproduce yarn force equilibrium, contact friction, cross-sectional compression, or relaxation recovery during the knitting process. Instead, under the assumptions of an ideal flexible yarn and geometry-driven modeling, priority is given to topological correctness, scale controllability, path continuity, meshability, and reproducible geometric input for polymer-fiber structure–property simulation. The implementation tools, including Python 3.10, Blender 5.0, and COMSOL 6.3, are only used for algorithm realization and numerical validation, while the methodological contribution lies in the rule-driven geometric generation strategy.

First, the basic knitting operations in weft-knitted structures are abstracted as a knitting-action set:

(1) $$A=\left\{K,T,F,P,B\right\}$$

where *K* denotes the knit action, *T* denotes the tuck action, *F* denotes the float action, *P* denotes the plating action, and *B* denotes front/back needle-bed assignment or bed-transfer action.

For single-bed structures, the pattern matrix *P_r_*_,*c*_ is used to describe the basic action of the needle at row *r* and column *c*. For plated structures, an additional plating marker *A_r_*_,*c*_ is introduced to generate an auxiliary yarn path without changing the basic action judgment. For double-bed structures, a bed-position symbol *b_c_* is further introduced to characterize front/back bed ownership, inter-bed offset, and cross-bed connection relationships.

**Figure 1:**
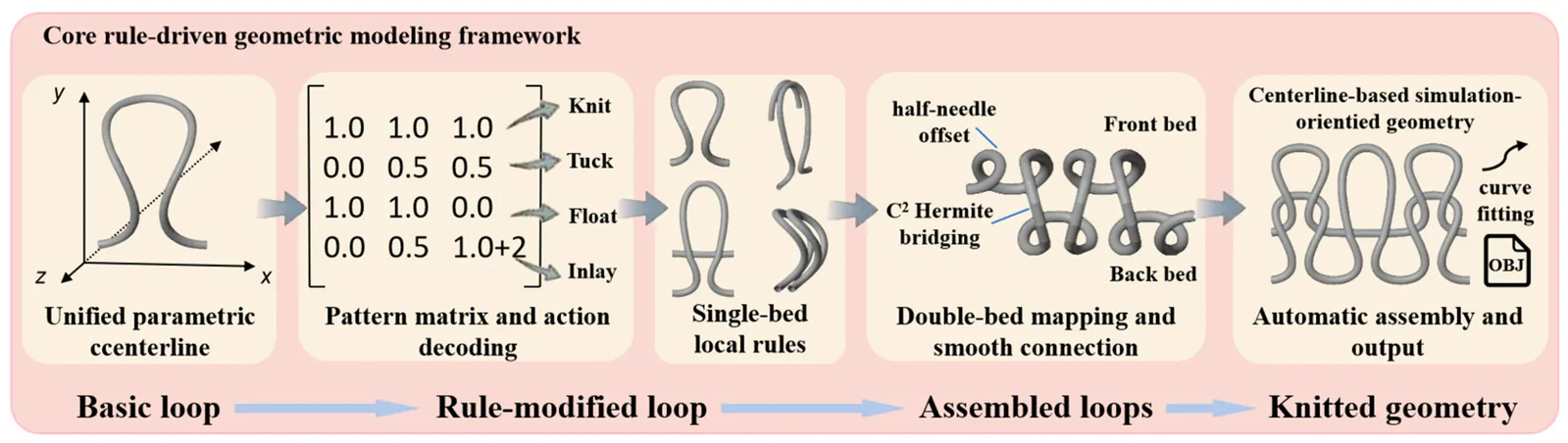
Algorithm workflow of the proposed rule-driven geometric modeling method for simulation-oriented knitted geometry generation.

The centerline-generation process is jointly constrained by the topological connection rule *R_topo_*, spatial displacement rule *R_space_*, and local deformation rule *R_deform_*, which can be expressed as

(2) $$\left\{P_{r,c},A_{r,c},b_{c}\right\}\to \left\{R_{topo},R_{space},R_{deform}\right\}\to \gamma \left(t\right)$$

where *γ*(*t*) is the finally generated yarn centerline. The topological rule determines the connection relationships among adjacent needles, adjacent courses, and front/back needle beds. The spatial rule defines the coordinate position of the centerline in the course, wale, and thickness directions. The local deformation rule describes local geometric responses such as tuck superposition, float-induced pulling, sinker-loop deepening, plating offset, and cross-bed transition. The algorithmic workflow of the proposed rule-driven geometric modeling method is illustrated in Figure 1. The algorithm converts knitting semantics into geometric operations through a sequential procedure, including structural input, action recognition, rule-based modification, topology reconstruction, continuity correction, and simulation-oriented geometry output.

Algorithm 1 summarizes the complete computational procedure of the proposed method. Different from conventional curve-fitting approaches, the proposed algorithm does not reconstruct each knitted structure independently. Instead, it generates different architectures by applying predefined geometric rules to a unified yarn-centerline representation.
**Algorithm 1. Rule-driven geometric modeling procedure for polymer-fiber knitted structures****Step****Procedure****Description**InputDefine modeling informationInput knitting pattern matrix *P*, knitting action set *A* = {*K*, *T*, *F*, *P*, *B*}, geometric parameter vector θ = {*a*, *h*, *d*, *rowOffset*}, and yarn radius r.1Initialize basic yarn representationGenerate the initial yarn centerline *γ*(*t*) using the proposed parametric centerline equations.2Decode knitting actionsAnalyze each element *P*(i,j) in the pattern matrix and identify the corresponding knitting action, including knit, tuck, float, plating, and bed assignment.3Apply action-based geometric rulesSelect the corresponding local deformation rule R(A(i,j)) and modify the basic centerline according to knitting actions and structural constraints.4Generate stitch-level trajectoriesConstruct individual stitch-level yarn paths by applying local geometric transformations, including loop extension, tuck deformation, float influence, plating offset, and double-bed displacement.5Assemble continuous yarn geometryConnect adjacent yarn segments using topology constraints and Hermite-based continuity correction to ensure C^1^ geometric continuity.6Perform geometric stability evaluationDetect potential geometric conflicts, including yarn intersection, excessive curvature variation, insufficient spacing, and local overlap.7Generate solid yarn geometryAssign yarn cross-section and reconstruct three-dimensional yarn solids through sweep-based modeling along the generated centerlines.8Export simulation-oriented modelConvert the reconstructed geometry into standardized three-dimensional geometry files for subsequent mesh generation and multiphysics simulation.OutputObtain simulation-readyOutput the final simulation-oriented knitted geometry G.

### 2.2. Parametric Yarn Centerline Representation

Under the above rule framework, a single loop is represented as a periodic three-dimensional parametric curve, and the whole fabric is formed by repeating, locally modifying, and continuously stitching this centerline unit in the course and wale directions according to structural rules. A right-handed Cartesian coordinate system is established, where the x-axis denotes the course direction, the y-axis denotes the wale direction, and the z-axis denotes the thickness direction. The basic loop centerline is defined as

(3) $$x\left(t\right)=t+a\cdot \sin\left(2t\right)$$

(4) $$y\left(t\right)=h\cdot \cos\left(t\right)$$

(5) $$z\left(t\right)=d\cdot \cos\left(2t\right)$$

(6) $$y_{row}\left(t\right)=h\cdot \cos\left(t\right)+rowOffset\cdot row$$

where *t* is a dimensionless periodic parameter used to describe the position evolution of the yarn centerline within one complete loop cycle. In this study, *t* varies from 0 to 2π, where one complete period corresponds to one basic knitted loop. The parameter *t* does not directly represent physical length; instead, it provides a normalized coordinate for describing the geometric transition of the loop. The actual loop dimensions are determined by the geometric coefficients *a*, *h*, and *d*, which have length dimensions and control the spatial amplitude of the yarn trajectory. Specifically, when t approaches 0 or 2π, *y*(*t*) reaches its maximum value, corresponding to the upper region of the loop (needle loop arc). When t approaches π, *y*(*t*) reaches its minimum value, representing the lower region of the loop (sinker loop arc). The two intervals *t* ∈ (0, π) and *t* ∈ (π, 2π) describe the transition regions between the upper and lower parts of the loop, corresponding to the two side connecting segments (loop legs). Therefore, the periodic formulation enables the complete loop geometry to be represented by a continuous and differentiable curve within one parameter cycle. *a* is the lateral expansion amplitude of the loop, mainly controlling loop width and transverse pore opening; *h* is the loop-height amplitude, used to control loop height and sinker-loop depth; *d* is the thickness-direction undulation amplitude, used to describe out-of-plane swing and interlayer separation; and *rowOffset* is the longitudinal offset between adjacent courses. These parameters collectively determine the physical morphology and spatial arrangement of knitted loops. The linear term *t* ensures the progressive evolution of the yarn path along the course direction, whereas the trigonometric terms generate periodic deformation and maintain geometric continuity. Although *t* itself is dimensionless, it establishes the normalized geometric sequence of the loop, while the dimensional parameters *a*, *h*, *d*, and *rowOffset* scale the generated geometry into the actual knitted structure. After being discretized with a fixed number of sampling points, the generated centerline can be further used for curve fitting, cross-section sweeping, solid modeling, mesh generation, and multiphysics performance simulation.

**Figure 2:**
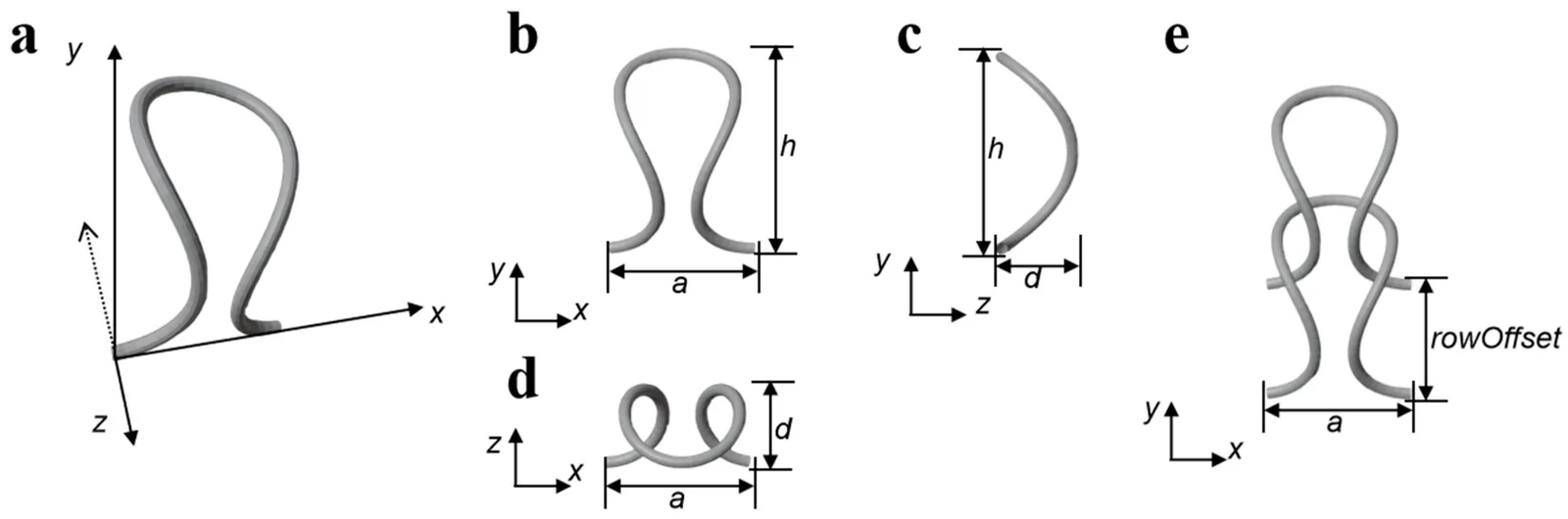
Regularized loop centerline and geometric-parameter definition: (**a**) three-dimensional centerline; (**b**) x-y plane; (**c**) z-y plane; (**d**) x-z plane; (**e**) inter-course offset.

To improve the understanding of the proposed geometric representation, the parametric deformation process of the yarn centerline is illustrated in Figure 2. Instead of directly constructing knitted fabrics using manually defined control points or interpolated curves, the proposed method first establishes a unified mathematical description of a basic yarn trajectory. The yarn centerline is controlled by several geometric parameters, including loop amplitude, loop height, thickness-related deformation, and spatial offset. By adjusting these parameters, different loop morphologies and local yarn configurations can be generated from the same initial centerline representation. As shown in Figure 2, the basic yarn path can be continuously transformed into various loop structures while maintaining geometric continuity. This parameterized representation provides a common geometric basis for subsequent knitting-action operations. Different knitting behaviors, such as knit, tuck, float, and inlay, are generated by applying local modification rules to the basic centerline rather than rebuilding independent geometric templates. Therefore, the proposed approach improves the scalability of geometric modeling for complex knitted structures. This centerline representation serves as the geometric foundation of Algorithm 1, where different knitting actions are realized through local modifications rather than independent geometric reconstruction.

### 2.3. Knitting-Action Encoding and Rule-Based Geometric Transformation

In the proposed algorithm, knitting structures are first converted into digital action information. Each action code corresponds to a predefined geometric transformation rule. For single-bed weft-knitted structures, the model calls the corresponding local geometric rule according to the action code of *P_r_*_,*c*_. Knit units are generated using the basic periodic centerline and serve as the main load-bearing units of the structure. When tuck or float actions, both non-knit actions, occur continuously above a knit unit, the upper arch of the underlying knit unit is moderately extended along the *y* direction to equivalently describe cross-course pulling. When local tuck superposition or an excessively small distance between adjacent paths occurs, the sinker-loop region is locally deepened in the *z* direction to represent layering and inward embedding effects.

A tuck unit is not modeled as a complete loop; instead, it is represented as a suspended arc supported by the underlying knit loop. Its lateral range is determined by the *x*-direction boundary of the supporting knit loop in the same column and is expanded by a margin coefficient. Its longitudinal height is constrained by the highest point of the nearest supporting knit loop below, and its thickness position is corrected according to the tuck layer. For continuous tuck groups, an intra-group platforming strategy and end smoothing are used to avoid local height oscillations caused by the superposition of multiple independent arches.

In this model, a float unit is regarded as a non-knit state and does not explicitly output an independent three-dimensional yarn segment. Instead, it affects the surrounding loops through a neighborhood-propagation mechanism. Specifically, the float action participates in the statistics of consecutive non-knit layers and further acts on the vertical extension and sinker-loop deepening of the underlying knit loop. This treatment can reduce the risk of yarn-path interpenetration, thickness overlap, and mesh-generation failure in complex patterns, while retaining the main geometric influence of float segments on neighboring morphology. A plated structure is generated by creating a second offset centerline based on the main-yarn centerline. The plated yarn path maintains the same topology and local correction rules as the main yarn, but a small spatial offset is applied to avoid complete overlap between the main and plated yarns. These rules represent the transformation mechanism between knitting semantics and three-dimensional yarn geometry.

### 2.4. Topological Assembly and Continuity Control

The knit, tuck, float, and plating actions in complex patterns are not independent of each other. Instead, the final geometry is jointly determined by vertical support relationships, spatial constraints between adjacent needles, and within-course path continuity. To avoid sharp corners, breakage, local oscillation, self-intersection, and thickness overlap, this study adopts strategies such as support constraints, neighborhood propagation, stabilization of continuous tuck groups, and action decoupling for local conflict treatment.

During within-course stitching, adjacent action units are sequentially connected according to needle position. When direct endpoint connection may cause a clear corner or tangent discontinuity, a cubic Hermite smoothing segment is introduced between adjacent units. Let the endpoint of the previous unit be *P*_0_ and the starting point of the next unit be *P*_1_, with corresponding endpoint tangent vectors *T*_0_ and *T*_1_. The connecting curve is then determined by the endpoint positions and local tangents, ensuring positional and tangential continuity at the connection. This treatment improves the smoothness of the yarn path and enhances the stability of subsequent sweep-based solid modeling and mesh generation. After local geometric transformation, the generated yarn segments are assembled according to topological relationships. Continuity constraints are introduced to maintain a physically reasonable and simulation-compatible yarn path.

### 2.5. Extension to Complex Knitted Architectures

Based on the single-bed structural rules, the present method is further extended to double-bed weft-knitted structures through needle-bed mapping, bed-position offset, and cross-bed connection rules. Each needle column is assigned a bed-position symbol *b_c_* = +1 or *b_c_* = −1, representing the front and back needle beds, respectively. To express the staggered needle arrangement of double-bed knitting, the back needle bed is shifted by half a needle pitch relative to the front bed in the x direction. In the thickness direction, the bed-position symbol also controls the out-of-plane undulation sign and the front/back bed-spacing offset:

(7) $$z(t)=b_{c}d\cos\left(2t\right)+b_{c}\frac{z_{gap}}{2}$$

where *z_gap_* is the reference spacing between the front and back needle beds. For the same knitting action, the front and back beds follow the same generation logic in the *x* and *y* directions, while a mirrored response is formed in the z direction through the bed-position symbol. When bed switching occurs between adjacent centerline segments, the model searches candidate cutting points near the ends of the two curves and selects the optimal combination based on tangential consistency and local turning characteristics. A Hermite smoothing segment is then constructed, and longitudinal and thickness-direction corrections are introduced to maintain continuity and clear interlayer relationships during cross-bed transition, thereby avoiding nonphysical interpenetration at front/back bed connections. The double-bed extension demonstrates the scalability of the proposed algorithm from simple single-bed structures to complex multi-layer knitted architectures.

### 2.6. Geometry Output and Simulation Preparation

After the geometric reconstruction process, the generated yarn centerlines are converted into standardized simulation-ready geometries. Parameter scanning is considered an application of the proposed modeling method for structure–property analysis. To analyze the influence of geometric parameters on fabric morphology and provide unified geometric input for subsequent batch simulation, a parameter-scanning mechanism is introduced. The main scanning parameters include the lateral expansion amplitude *a*, loop height *h*, thickness-direction undulation *d*, and course spacing *rowOffset*. Each parameter is defined by a starting value, ending value, and step size. When the step size is less than or equal to zero, the parameter is treated as a fixed input. For any parameter combination, the model executes the same process of action recognition, centerline generation, local correction, continuous stitching, and standardized output, without redefining curve templates for different structures.

The finally generated yarn centerlines are output as standardized geometric files for subsequent cross-section sweeping, solid reconstruction, mesh generation, and multiphysics numerical simulation. To improve reproducibility, the same action-recognition rules, parametric centerline equations, stitching strategy, and output format are used for all structures and parameter combinations. This workflow transforms knitting-structure semantics into computable three-dimensional geometric input, providing a stable and reusable modeling basis for structural-parameter screening, structure–property analysis, and moisture-transfer simulation of polymer-fiber knitted structures.

In the present work, the computational details required for reproducing the geometry-generation process include the pattern matrix; knitting-action code; geometric parameters *a*, *h*, *d*, and *rowOffset*; sampling density; yarn radius used for solid sweeping; and mesh-quality indicators. These items were kept consistent when comparing the proposed method with the interpolated-curve method, so that differences in meshability and simulation suitability mainly arise from the generated yarn-path geometry rather than from inconsistent preprocessing settings.

### 2.7. Evaluation of Geometric Modeling Quality

To evaluate the structural generality of the proposed modeling framework, several representative knitted architectures were selected as benchmark cases, including plain knit, tuck, plated, rib, interlock, cardigan, and purl structures. These structures contain different knitting actions and topological relationships, allowing evaluation of the capability of the proposed method in handling single-bed, double-bed, and multi-yarn configurations.

To avoid judging the modeling result only by three-dimensional appearance, the computability of the model is further evaluated from the perspective of centerline geometric stability. The evaluation indicators include the number of centerline points, minimum path distance, curvature-jump rate, maximum bending angle, and mean error of key points. Among them, the number of centerline points characterizes the discretized representation of the path. The minimum path distance is used to identify whether adjacent discontinuous yarn paths are too close, overlapped, or at risk of potential interference. The curvature-jump rate and maximum bending angle are used to evaluate local path smoothness and bending risk. The mean error of key points is used to compare the consistency of different modeling methods at major geometric-feature positions.

During calculation, the minimum path distance is obtained from non-adjacent path-point distances after excluding adjacent points on the same centerline. The curvature-jump rate is expressed as the proportion of points at which the included angle between adjacent line segments exceeds a specified threshold. The mean error of key points is calculated by selecting the start point, endpoint, and extreme points in the *x*, *y*, and *z* directions. Before comparison, bounding-box scale alignment and necessary coordinate-direction correction are performed. These indicators supplement the assessment of whether the model is suitable for subsequent solid sweeping, mesh generation, and numerical solution from three aspects: path spacing, local continuity, and geometric-position consistency. These indicators evaluate whether the generated geometry satisfies the requirements of numerical simulation preprocessing.

### 2.8. Fabric Moisture-Transfer Simulation and Experimental Validation

To verify the applicability of the constructed polymer-fiber knitted-structure models in the preprocessing stage of numerical simulation and to evaluate their meshability under complex spatial topology, typical structures are further subjected to solid modeling, mesh generation, and mesh-quality statistical analysis after the yarn-centerline model is established. After importing the fabric centerline coordinate points into the simulation software, yarn solids are formed by cross-section sweeping, and three-dimensional mesh generation is performed. During mesh generation, the overall view and locally enlarged regions of different structural models are examined, with emphasis on mesh conformity and adaptability in high-curvature loop-head regions, transition regions between loop columns and curved segments, and regions where adjacent yarn paths are spatially close. After mesh generation, the resulting mesh is further statistically evaluated. To comprehensively characterize mesh quality, the minimum element quality and average element quality are used as overall indicators, and quality parameters such as skewness, maximum angle, condition number, growth rate, and curved skewness are combined to quantitatively compare meshes generated by different modeling methods. This evaluation links geometric modeling quality with the reliability of subsequent numerical analysis of polymeric fibrous materials.

A fully plated plain-knitted polymer-fiber fabric model is constructed using the proposed regularized geometric modeling method and is then used for multiphysics coupled calculation in COMSOL. The fully plated plain-knitted structure introduces a spatially parallel relationship between the main yarn and the plated yarn on the basis of the basic plain-knitted structure. This configuration can form relatively clear thickness-direction covering differences and wetting-transport differences, thus exhibiting distinct unidirectional moisture-transfer characteristics. To reduce computational cost and improve solution efficiency, the minimum structural repeat unit of the fabric is selected as the computational domain. The level-set two-phase flow method is used to describe the dynamic evolution of the droplet–air interface, and a porous-medium model is combined to represent liquid seepage inside the yarn. Under room temperature and standard atmospheric pressure, the density and dynamic viscosity of water (998 kg/m^3^ and 1.0 × 10^−3^ Pa·s) and air (1.2 kg/m^3^ and 1.8 × 10^−5^ Pa·s) were obtained from the built-in material property database of COMSOL Multiphysics and previous fluid-property studies [29,30,31]. The water–air surface tension coefficient was set as 0.072 N/m, in line with reported experimental values [32].

To improve the reproducibility of the numerical simulation, the detailed COMSOL implementation settings are provided. The coupled moisture-transfer model was solved using COMSOL Multiphysics 6.3 with a time-dependent solver. The transient simulation was performed within a time range of 0–0.2 s using a fixed time step of 0.0001 s. The relative tolerance was set to 0.1 to ensure convergence of the transient solution.

The three-dimensional computational domain was discretized using free tetrahedral elements. A unified mesh strategy was adopted for all simulated structures, and the mesh was generated after yarn solid reconstruction through cross-section sweeping. The mesh quality was evaluated using element-quality indicators to ensure numerical stability during the coupled transport simulation.

For the multiphysics boundary conditions, the outer surface of the fabric model was treated as the liquid–air interaction boundary, where the initial droplet was introduced. The yarn interior was modeled as a porous medium to describe liquid infiltration, while the surrounding region was defined as an air domain. No-slip conditions were applied to solid yarn surfaces, and continuity conditions were maintained at the interfaces between different domains.

The material parameters considered in the present multiphysics simulation include fluid properties and polymer-fiber-related transport characteristics. The density and dynamic viscosity of water and air were obtained from standard thermophysical property databases. The yarn region was represented using a porous-medium approach, where the equivalent transport behavior was determined by the structural characteristics of the yarn assembly. Since the primary objective of this work is to establish a geometry-generation framework rather than a polymer-specific constitutive model, the polymer-dependent properties can be replaced according to different fiber systems in future simulations. These parameters were kept consistent for all simulations.

The polymer-fiber knitted fabric used for experimental validation was the plated structure described above, consisting of a face yarn of 75D/144F and a ground yarn of 75D/12F. The plated configuration was selected because the two yarn systems form different surface characteristics between the two sides of the fabric, making it suitable for evaluating directional moisture transport performance. The purpose of this experiment was to verify whether the simulation-oriented geometric model can provide reliable geometric input for predicting the moisture-transfer behavior of polymer-fiber knitted structures.

The moisture-management performance of the fabric was evaluated using a moisture management tester (M290, SDL Atlas, Rock Hill, SC, USA) according to the reported testing procedure [33,34]. The tested specimens were circular polymer-fiber knitted fabrics with a diameter of 9 cm. Before testing, all specimens were conditioned under standard atmospheric conditions at room temperature for 24 h to minimize the influence of environmental variations. A 0.9 wt% sodium chloride solution was prepared to simulate human sweat. During each measurement, 0.22 mL of the solution was delivered onto the fabric surface, and the total delivery time of the liquid was controlled at 20 s. The moisture-transfer response was continuously recorded for 120 s.

In this study, the surface exposed to air was defined as the face side, whereas the surface in contact with skin was defined as the back side. Measurements were performed separately with the face and back sides placed upward. The moisture-transfer signals and one-way liquid transport capability were obtained from the recorded voltage curves. The experimental results were subsequently compared with the simulation results to evaluate the accuracy and applicability of the proposed simulation-oriented modeling approach. To ensure the reproducibility of the experimental validation, one representative fabric specimen was prepared and measured three times under identical testing conditions according to the AATCC TM195-2011 standard [35]. The reported moisture-transfer parameters were calculated as the average values of the repeated measurements. The test was conducted with the front and back sides of the fabric facing up, respectively. The conduction data of moisture inside the fabric and the one-way transport index were obtained from the voltage signal curve.

The simulation results yielded the following:

(8) $$S_{sim}=\int_{A}\varphi \left(x,y,z\right)dA$$

where *ϕ*(*x*,*y*,*z*) denotes the liquid volume fraction, and *dA* denotes the surface area element (m^2^). The total water absorption volume was then obtained according to Equation (9):

(9) $$V_{liq}=S_{sim}\cdot d$$

where *d* denotes the thickness of the cross-section (m). The water absorption capacity was further obtained according to Equation (10):

(10) $$M_{liq}=V_{liq}\cdot \rho =S_{sim}\cdot d\cdot \rho$$

where *ρ* denotes the liquid density (1000 kg/m^3^). The one-way transport index was finally obtained according to Equation (11):

(11) $$O=M_{B}-M_{T}/t$$

where *M_B_* denotes the water absorption capacity of the lower surface, *M_T_* denotes the water absorption capacity of the upper surface, and *t* denotes time (s). According to Equation (11), the one-way liquid transport indices under different liquid supply directions were calculated from the surface-dependent water absorption capacities. When liquid was supplied from the face side, the corresponding indices were denoted as OF and OF’, whereas OS and OS’ represented the indices obtained when liquid was supplied from the back side. The simulated values were calculated using the same definition by replacing the experimentally measured absorption capacities with the COMSOL-derived water absorption capacities.

To evaluate the repeatability of the experimental measurements, each fabric specimen was tested repeatedly under identical conditions. Three independent measurements were performed for each sample, and the obtained moisture-transfer parameters were expressed as the mean value with standard deviation (SD). The 95% confidence interval (CI) was calculated based on the Student’s t-distribution to quantify the uncertainty of the experimental measurements. The statistical consistency between the simulated and experimental results was evaluated by comparing the mean experimental values and the corresponding confidence intervals. Since the main purpose of the experimental validation was to verify the feasibility of the proposed geometric modeling framework rather than to establish a statistical prediction model, the relative error between simulation and experiment was used as the primary accuracy indicator.

## 3. Results

### 3.1. Analysis of Structural Representation Capability

**Figure 3:**
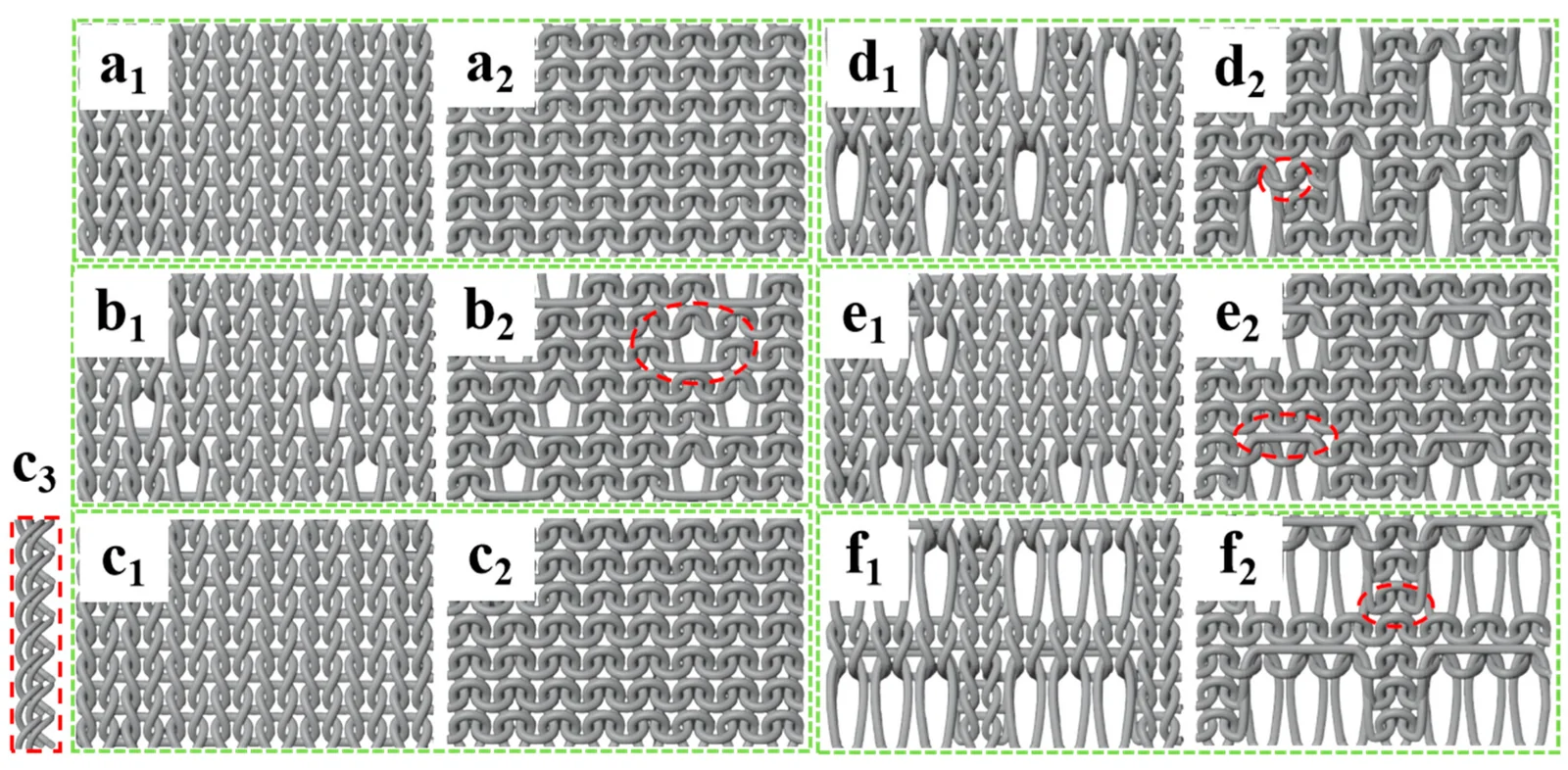
Modeling results of single-bed polymer-fiber weft-knitted structures: (**a**) plain stitch; (**b**) jacquard; (**c**) plated structure; (**d**) single-needle double-course tuck; (**e**) double-needle single-course tuck; (**f**) three-needle double-course tuck. Labels 1 and 2 denote the face and back sides of the fabric, respectively, and label 3 denotes the side view. The red ring represents the position of the settlement arc change. The red rectangle represents the position of adding yarn.

To further evaluate the general applicability of the proposed modeling framework, multiple representative knitted structures were generated and analyzed, covering typical single-bed and double-bed knitting configurations. First, the most basic plain-knitted fabric was selected for three-dimensional geometric modeling among single-bed weft-knitted structures, as shown in Figure 3(a_1_,a_2_). The results show that the established rule-driven modeling framework can accurately reconstruct the periodic arrangement of loop units in the course and wale directions in plain-knitted structures and can reasonably reflect the basic face/back hierarchy of the fabric and the intermeshing relationship between loops. Meanwhile, the yarn path inside the loop unit changes smoothly without obvious sharp turning. At the connection positions between adjacent units, the curve transition is natural, and local tangential variation is continuous, indicating that the method can maintain good overall geometric consistency when representing basic structures. This demonstrates that the rule-driven modeling method can not only correctly recognize the topology of basic weft-knitted structures but also provide a geometric basis for subsequent simulation of polymeric fibrous materials.

On this basis, the ability of the proposed method to represent complex single-bed structures with local structural variation is further examined. Figure 3(b_1_,b_2_) shows the modeling result of a jacquard structure. According to the structural actions corresponding to different regions in the pattern matrix, the model can automatically generate surface morphology and structural distribution features with regional differences, as shown in Figure 3(b_1_). Further observation shows that, while maintaining the correctness of the overall topology, the model captures changes in local loop geometry and surface hierarchy. The degree of loop stretching and structural undulation in different regions changes according to the rule input without causing overall structural breakage or unreasonable stitching, as indicated by the red loop region in Figure 3(b_2_). This result indicates that the proposed method can establish an effective mapping between local structural actions and local geometric deformation.

In addition to local pattern variation, this study further validates the ability of the method to represent multi-yarn polymer-fiber structures. Figure 3(c_1_,c_2_,c_3_) shows the modeling result of a plated structure. Within the unified parametric framework, the model can not only represent the basic knitted structure formed by the main yarn, as shown in Figure 3(c_1_,c_2_), but can also embed an additional yarn into specified structural positions according to preset rules, thereby constructing a hierarchical spatial structure consisting of both the main yarn and the plated yarn, as shown in Figure 3(c_3_). This result indicates that the proposed method can effectively describe the relative configuration of multiple yarn systems in plated structures and reflects the adaptability of the unified rule framework to polymeric multi-component knitted structures.

To further examine the capability of the model to represent complex knitting actions, single-bed structures under different tuck conditions are modeled, as shown in Figure 3(d_1_,d_2_,e_1_,e_2_,f_1_,f_2_), corresponding to single-needle double-course tuck, double-needle single-course tuck, and three-needle double-course tuck structures, respectively. As the number of tuck needles and the number of continuous tuck courses change, the model clearly reflects differences in cross-needle connection, loop stretching morphology, pore distribution, and surface hierarchy of local structural units, as shown in the red loop regions in Figure 3(d_2_,e_2_,f_2_). Notably, the single-needle, double-needle, and three-needle tuck structures can all be uniformly represented within the same rule system, and their differences are mainly distinguished by changes in local path span, upper suspended-arc range, and structural surface features. These results indicate that the method is applicable not only to basic single-bed structures but also to the geometric mapping of different local knitting actions in complex single-bed structures within a unified rule framework, showing strong structural adaptability and representation capability.

**Figure 4:**
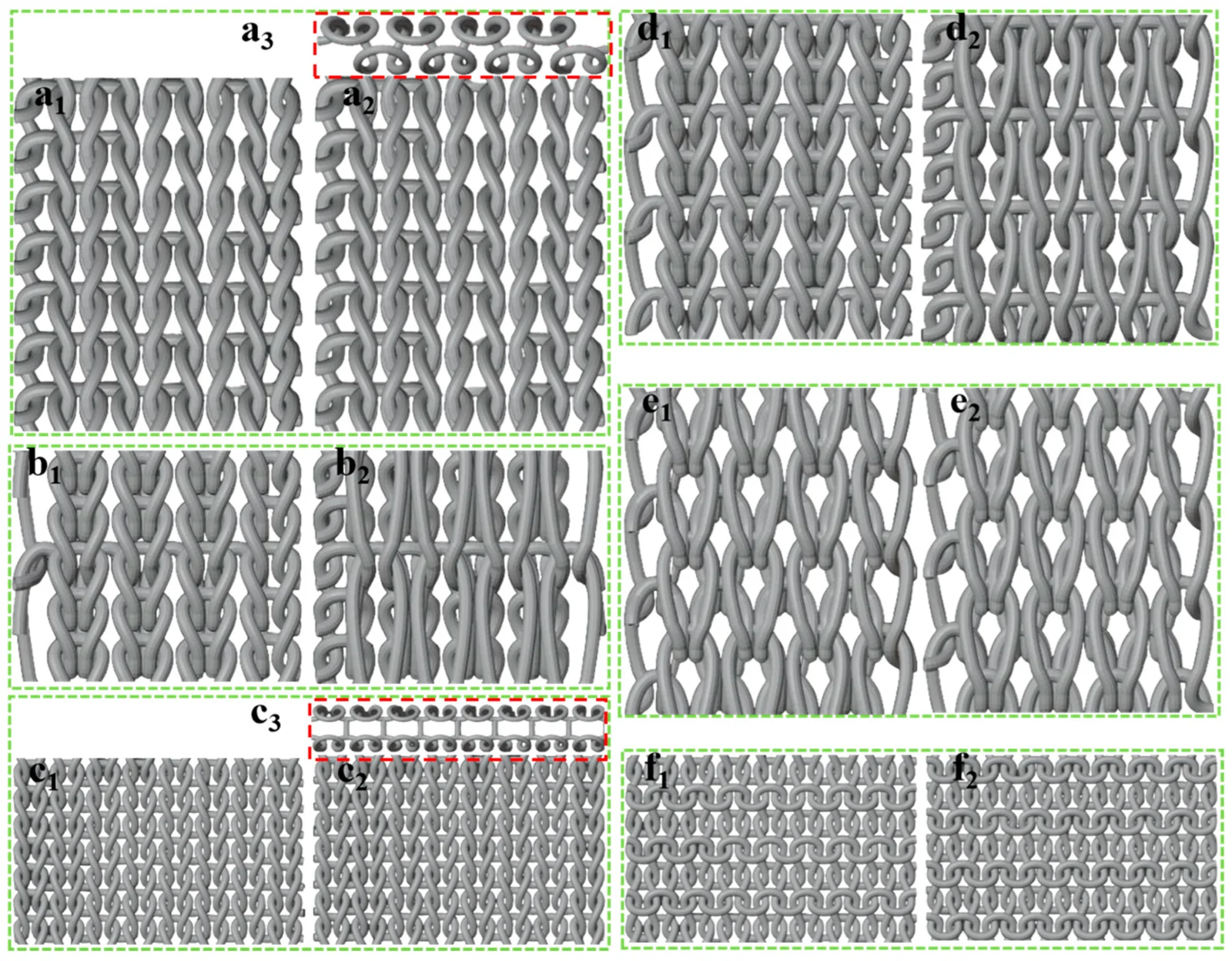
Modeling results of double-bed polymer-fiber weft-knitted structures: (**a**) 1 + 1 rib; (**b**) interlock; (**c**) half cardigan; (**d**) self-designed double-face tuck fabric; (**e**) full cardigan; (**f**) purl structure. Labels 1 and 2 denote the face and back sides of the fabric, respectively, and label 3 denotes the side view. The red rectangle represents the position of the front and rear needle beds.

After validating single-bed structures, the method is further extended to double-bed structures to examine its structural recognition and geometric representation capability for double-face and complex double-bed polymer-fiber weft-knitted structures. Figure 4 presents the three-dimensional modeling results of representative structures, including 1 + 1 rib, interlock, half-cardigan, self-designed double-face tuck fabric, full-cardigan, and purl structures. For basic double-bed structures such as 1 + 1 rib and interlock, as shown in Figure 4(a_1_,a_2_,a_3_,b_1_,b_2_), the model can accurately reproduce the double-layer structural features formed by alternating front- and back-bed knitting and can reasonably represent the front/back bed assignment and interlayer configuration of double-bed structures. This indicates that the method can maintain high topological-recognition accuracy and spatial-representation consistency for basic double-bed structures. For typical double-bed structures with more-pronounced surface textures and hierarchical differences, such as the half-cardigan, full-cardigan, and purl structures shown in Figure 4(c_1_,c_2_,c_3_,e_1_,e_2_,f_1_,f_2_), the model effectively reflects the differences in spatial arrangement, surface texture, and thickness-direction layer distribution of loop units under different bed configurations. This demonstrates that the proposed method can not only recognize the front/back bed assignment but also effectively represent geometric-feature variations caused by bed configuration differences in double-bed structures.

On this basis, a self-designed double-face tuck fabric was further selected for validation, as shown in Figure 4(d_1_,d_2_). Compared with the previous basic double-bed structures, this structure is more complex in terms of local action combinations and front/back bed connection relationships. Nevertheless, the modeling results show that the model can still uniformly represent the structural units and their connection relationships in the front and back needle beds according to the rule-recognition results, while maintaining good topological integrity and clear hierarchy. The above results indicate that all structures were successfully reconstructed using the same rule-driven framework without manually redefining individual geometric templates. This demonstrates that the proposed method can transform different knitting actions into unified geometric generation rules.

### 3.2. Comparison of Modeling Methods and Analysis of Centerline Geometric Stability

**Figure 5:**
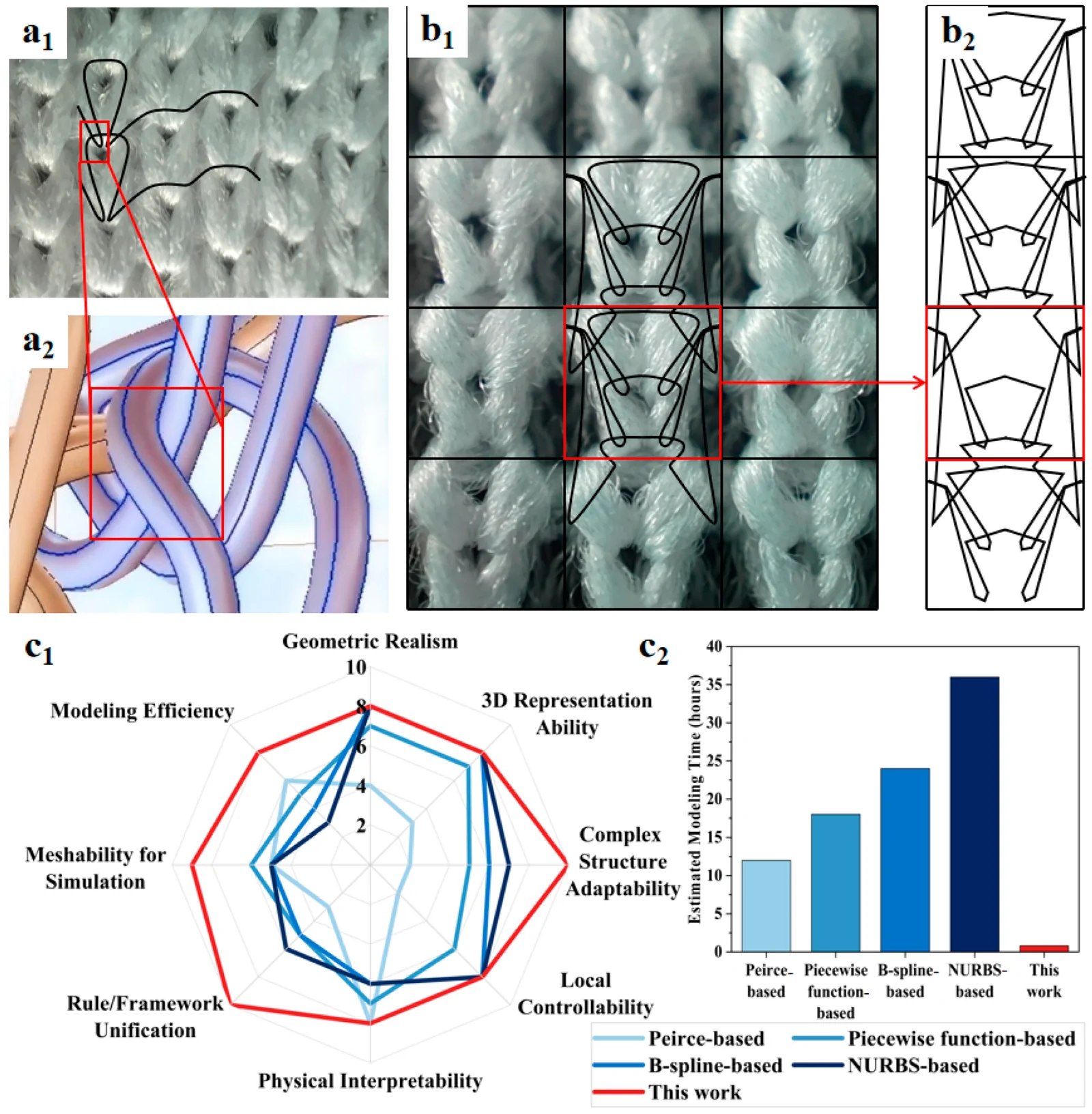
Preprocessing illustration of the interpolated-curve method for single-bed and double-face tuck fabrics and comprehensive comparison of different methods: (**a_1_**) abstraction of the centerline of a single-bed tuck fabric; (**a_2_**) local enlargement of the modeling result of the single-bed tuck fabric; (**b_1_**) abstraction of the centerline of a double-face tuck fabric; (**b_2_**) feature-point selection for the double-face tuck fabric; (**c_1_**) radar chart comparing the advantages of different modeling methods; (**c_2_**) comparison of modeling efficiency among different methods.

As shown in Figure 5, when the interpolated-curve method is used for geometric modeling of single-bed tuck and double-face tuck fabrics, the yarn centerline path must first be manually abstracted according to the morphology of the actual fabric. Figure 5(a_1_,b_1_) show that this method does not directly generate the structure from knitting rules. Instead, the modeler must manually determine key path directions and complete the abstract representation of the centerline based on the intermeshing relationship of loops, the floating and sinking states of yarns, and the spatial positions of front and back layers. Especially for double-face tuck structures, because the yarn paths on the front and back needle beds are more intricately interlaced and the local hierarchy is richer, feature points must be further selected and piecewise interpolation must be performed, as shown in Figure 5(b_2_). This process places high demands on the modeler’s knowledge of knitted structures, spatial imagination, and three-dimensional reconstruction experience, making the modeling process highly experience-dependent and subjective.

At the same time, the locally enlarged result in Figure 5(a_2_) indicates that although feature-point interpolation can reconstruct yarn paths to some extent, improper feature-point selection or insufficient local-transition control can easily cause path overlap, self-intersection, or local interpenetration in complex crossing regions. In particular, when the tuck suspended arc is close to the adjacent sinker loop, conventional control-point interpolation can easily produce excessive local bending, causing the swept solid to overlap or locally twist in the curved region and thereby reducing mesh quality. In the proposed method, local elevation after action judgment, supporting-loop height constraints, end smoothing, and neighborhood propagation are used to keep the suspended-arc transition region at a relatively reasonable spatial distance and reduce the risk of curvature jumps at the connection.

To further quantitatively illustrate the differences between the two modeling methods at the centerline-path level, the number of centerline points, minimum path distance, curvature-jump rate, maximum bending angle, and mean error of key points are calculated based on the centerline coordinates of four structures: plain stitch, fully plated plain stitch, single-needle double-course tuck, and double-face tuck. The results are listed in Table 3. The table shows that, for the four structures, the conventional interpolated-curve method requires fewer centerline points, but its curvature-jump rate and maximum bending angle are generally higher, indicating obvious direction changes at local path connections. For the plain-knitted structure, the curvature-jump rate and maximum bending angle of the interpolated-curve method are 40.78% and 82.53°, respectively, whereas those of the proposed method decrease to 0 and 10.39°, respectively. For the fully plated plain-knitted structure, while maintaining the spatial consistency between the main-yarn and plated-yarn paths, the proposed method reduces the curvature-jump rate from 61.54% to 0 and the maximum bending angle from 61.04° to 14.21°, with a mean key-point error of only 0.0131%.

For complex structures such as single-needle double-course tuck and double-face tuck, the curvature-jump rates of the conventional interpolated-curve method are 56.25% and 69.23%, respectively, and the maximum bending angles both exceed 110°, indicating that strong local corners are likely to occur in tuck suspended arcs, adjacent loop connections, and cross-bed transition regions. Through rules such as supporting-loop height constraints, local elevation, neighborhood propagation, bed mapping, and cross-bed continuous connection, the proposed method reduces the curvature-jump rates of these two structures to 0.31% and 0.26%, respectively, and reduces the maximum bending angles to 51.07° and 47.09%, respectively. It should be noted that the minimum path distance of the proposed method in complex tuck structures is relatively small, mainly because more suspended arcs, interlayer transitions, and neighborhood-deformation paths are retained. This indicator should be evaluated together with yarn radius and solid-sweeping results and can be further improved by adjusting local elevation or thickness-layering parameters.

To compare the proposed method with existing knitted fabric geometric modeling methods, Figure 5(c_1_,c_2_) present a comprehensive performance radar chart and a modeling-efficiency comparison, respectively. Existing methods can usually achieve acceptable geometric representation for basic structures. However, when dealing with complex polymer-fiber textile structures such as jacquard, plated, tuck, half-cardigan, and full-cardigan fabrics, especially complex double-bed structures, they often rely on manual feature-point selection, path segmentation, and spatial-shape abstraction, imposing high requirements on researchers’ knowledge of knitted structures and three-dimensional reconstruction experience. For double-bed structures, methods based on coordinate-point fitting or local curve stitching can easily cause path overlap, local interpenetration, or even self-intersection at the front/back needle-bed junction, thereby reducing geometric stability and further affecting the subsequent mesh-generation and numerical-simulation process. In contrast, the proposed method is driven by knitting actions and structural rules and directly generates three-dimensional yarn paths within a unified parametric framework. It reduces dependence on manual experience in complex-structure modeling while considering local-connection continuity, reasonable spatial hierarchy, and simulation-preprocessing suitability during path generation. As shown in Figure 5(c_1_), the proposed method exhibits clear comprehensive advantages in geometric realism, three-dimensional representation capability, adaptability to complex structures, rule unification, meshability, and modeling efficiency. Figure 5(c_2_) further shows that the method significantly reduces modeling time while maintaining the ability to represent complex structures, making it more suitable as a geometric basis for numerical simulation and structure–property analysis of polymer-fiber knitted materials.

### 3.3. Mesh-Generation Adaptability and Quality Analysis

**Figure 6:**
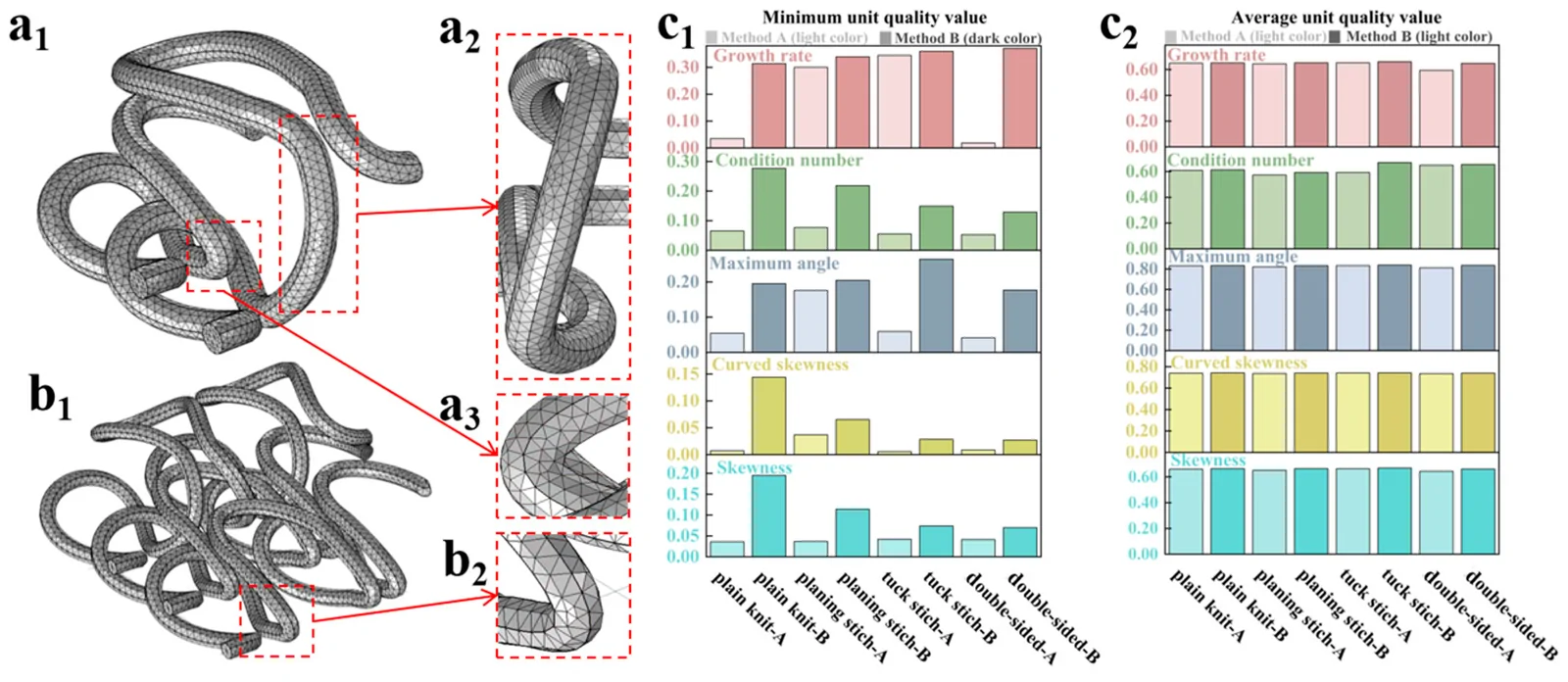
Fabric mesh-generation results and mesh-quality comparison of different modeling methods: (**a**) mesh-generation result of the half-cardigan fabric; (**b**) mesh-generation result of the full-cardigan fabric, where labels 1, 2, and 3 denote the overall view and locally enlarged views; (**c**) mesh-quality statistics of different modeling methods, where (**c_1_**) shows the minimum element quality and (**c_2_**) shows the average element quality.

Representative half-cardigan (Figure 6(a_1_,a_2_,a_3_)) and full-cardigan (Figure 6(b_1_,b_2_)) fabrics are selected for mesh-generation analysis after solid modeling. For these two complex polymer-fiber knitted structures with clear spatial hierarchy and locally close yarn paths, the surfaces of the generated yarn solids form continuous, complete, and relatively uniformly distributed triangular meshes. No obvious mesh loss, local collapse, or nonphysical overlap is observed. In regions with large loop-head curvature, transition regions between loop columns and curved segments, and regions where adjacent yarn paths are spatially close, the mesh can still conform well to the geometric boundary, and local element shapes remain relatively regular. This indicates that the geometric models generated by the proposed method have good geometric stability and preprocessing suitability and can provide a reliable geometric basis for subsequent numerical simulation. Figure 6(c_1_,c_2_) compares the mesh-quality statistics of the interpolated-curve method (method A) and the proposed method (method B). Figure 6(c_1_) shows the numerical comparison of minimum element quality, and Figure 6(c_2_) shows the numerical comparison of average element quality. The compared indicators include growth rate, condition number, maximum angle, curved skewness, and skewness. The results show that method B is generally superior to method A for all five indicators. For both minimum element quality and average element quality, the proposed method shows a higher quality level. This demonstrates that, compared with the interpolated-curve method, the proposed method can more effectively avoid local geometric abrupt changes, path interference, and element distortion during complex-structure modeling, leading to better stability and consistency in mesh generation.

Further analysis of specific mesh-quality indicators shows that the proposed method produces a more obvious improvement in locally poor-quality elements. Taking the plain-knitted structure as an example, for minimum element quality, the values of the interpolated-curve method in terms of skewness, curved skewness, maximum angle, condition number, and growth rate are 0.03575, 0.00727, 0.05433, 0.06526, and 0.03488, respectively. The corresponding values of the proposed method increase to 0.1952, 0.1441, 0.1952, 0.2767, and 0.3138, respectively. For the fully plated plain-knitted, single-needle double-course tuck, and for double-face tuck models containing additional yarns or complex transition structures, the proposed method also improves the minimum quality level. The average element-quality results also show that the proposed method maintains a relatively high overall mesh quality, with an average skewness quality of approximately 0.66, an average maximum-angle quality of approximately 0.83, and an average curved-skewness quality of approximately 0.74. These results indicate that the regularized modeling method can reduce low-quality elements caused by unevenly distributed discrete points, abrupt path changes, or local interference, thereby lowering the risk of mesh failure or solution instability in subsequent simulations.

### 3.4. Comparison Between Moisture-Transfer Simulation and Experimental Results

**Figure 7:**
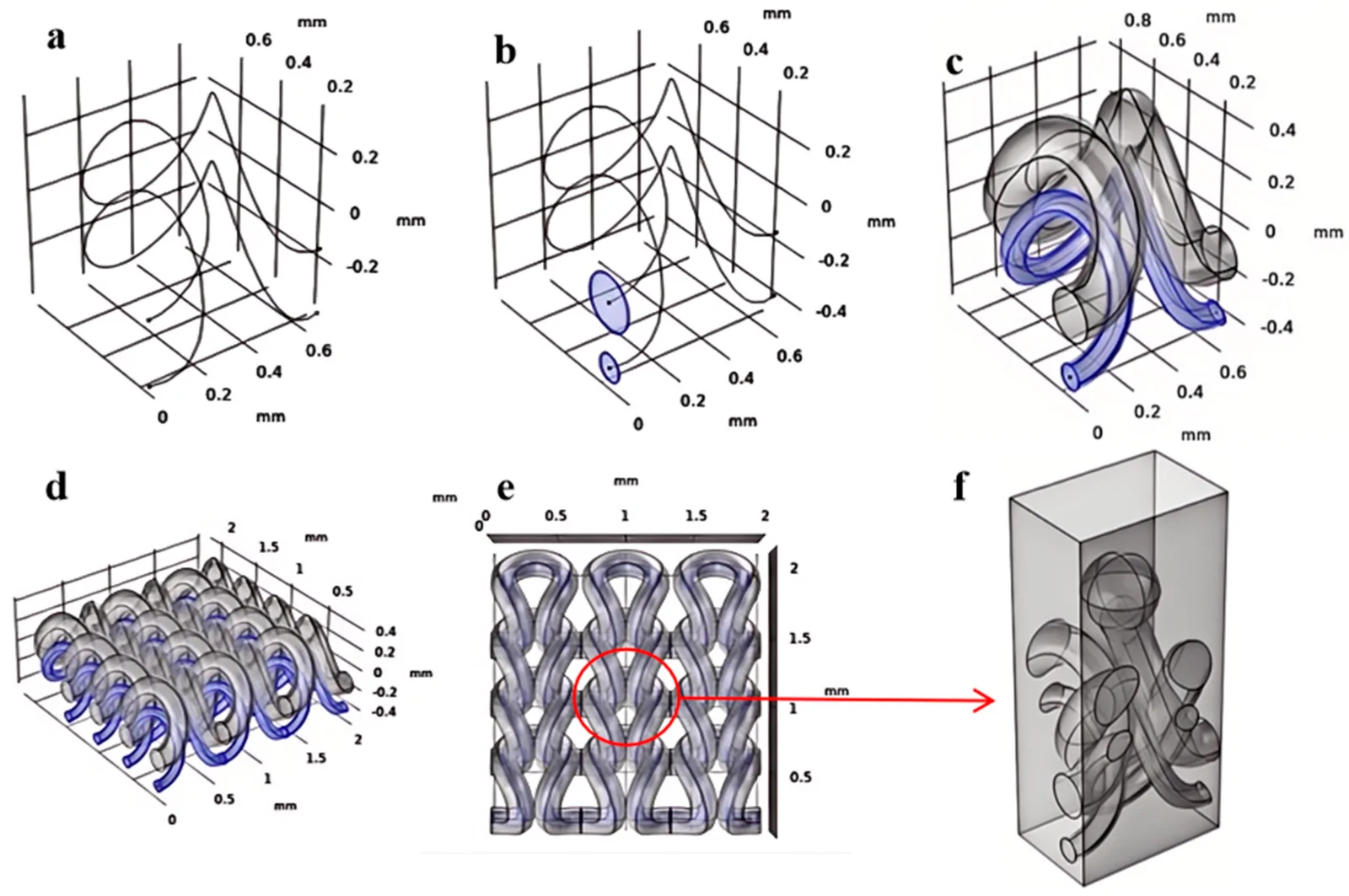
Model-import workflow for the fully plated plain-knit polymer-fiber fabric: (**a**) Coil centerline; (**b**) Sweeping curve; (**c**) A three-dimensional view of a coil; (**d**) Array 3 × 4 3D view; (**e**) Array 3 × 4 plan view; (**f**) Moisture simulation model.

Figure 7 shows the model-import process of the fully plated plain-knitted polymer-fiber fabric. The results indicate that the generated model can be smoothly introduced into the subsequent simulation workflow. The geometric boundary is clear, and the structural topology is well defined, supporting material-parameter assignment, boundary-condition application, and numerical solution. On this basis, unidirectional moisture-transfer simulation is conducted to evaluate whether the meshable yarn geometry can support performance prediction of a polymeric fibrous textile system.

**Figure 8:**
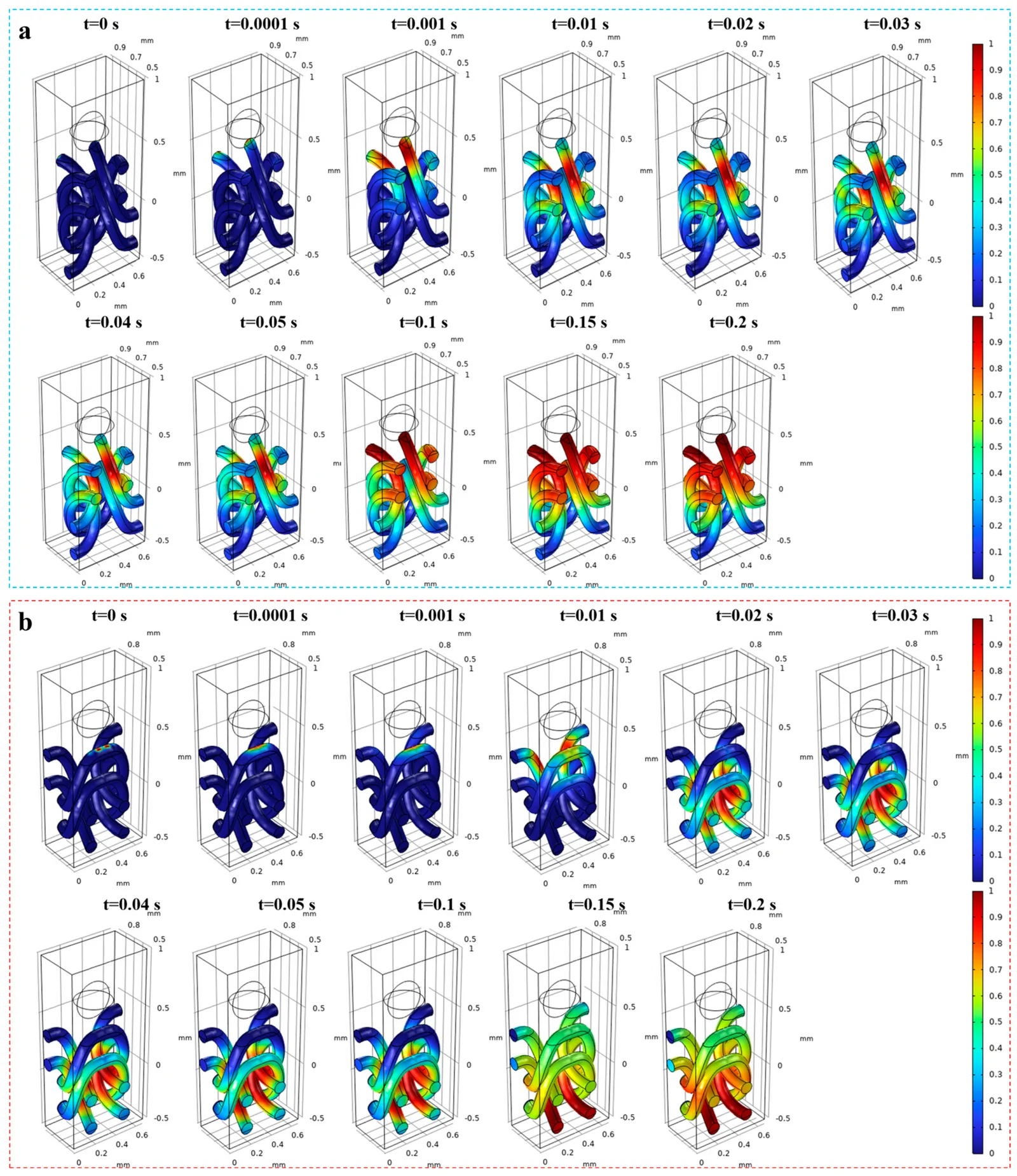
Liquid-content distribution contours of the plated fabric from 0 to 0.2 s: (**a**) droplets penetrating from the fabric face; (**b**) droplets penetrating from the fabric back.

Figure 8 presents the unidirectional moisture-transfer simulation results of the fully plated plain-knitted polymer-fiber fabric. When the droplet enters from the face side, it contacts the fabric and spreads over the upper surface. At steady state, the liquid content on the upper surface is higher than that on the lower surface, indicating that the droplet has not been transported to the lower fabric surface. When the droplet enters from the back side, it rapidly transports through the fabric thickness after contacting the fabric. At 0.02 s, the liquid has reached the lower surface and spreads along it. At steady state, the liquid content on the lower surface is higher than that on the upper surface, indicating that the liquid has been transported to the lower surface. These results show that the plated structure forms an asymmetric transport pathway and exhibits good unidirectional moisture-transfer behavior, which is consistent with the intended structure-performance relationship of moisture-management polymer textiles.

**Figure 9:**
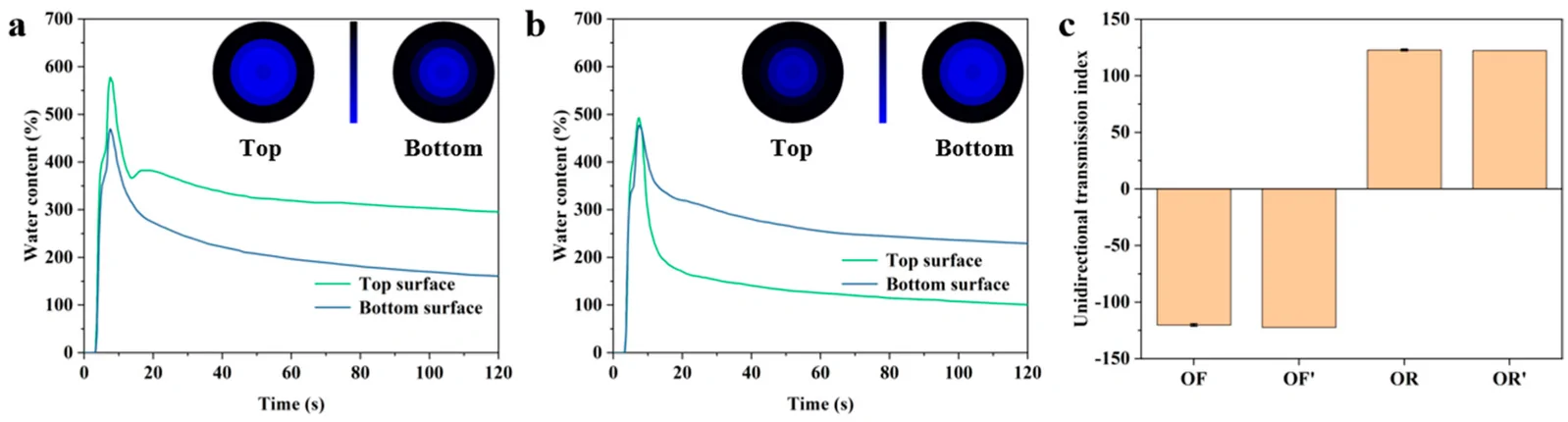
Experimental and simulated values of the one-way liquid transport capability on the face and back sides of the plated polymer-fiber fabric: (**a**) The test results when the front of the fabric is submerged in water; (**b**) The test results when the fabric is submerged in water on the opposite side; (**c**) Comparison between measured and simulated values of one-way transmission index

Figure 9 presents the experimental results of the plated polymer-fiber knitted fabric and the comparison between experimental measurements and simulation predictions. The experimental values were obtained from three repeated measurements under identical conditions and are presented as mean values. As summarized in Table 4, the mean one-way liquid transport capability values were −120.2257 for the face side and 122.8615 for the back side, with standard deviations of 1.0159 and 0.8855, respectively. The corresponding 95% confidence intervals indicate limited variation among repeated measurements, demonstrating good repeatability of the experimental procedure.

The simulated one-way liquid transport capability values were −122.3187 and 122.2472 for the face and back sides, respectively. Compared with the experimental mean values, the relative errors were 1.74% and 0.50%, respectively, indicating good agreement between the simulation predictions and experimental measurements. According to the performance classification standard of AATCC TM195-2011, both the experimental and simulated one-way liquid transport performances of the fabric were classified as Grade 3. These results demonstrate that the proposed geometry-generation method can provide reliable simulation-ready models for predicting directional moisture transport in polymer-fiber knitted textiles. However, further validation involving more polymer types, yarn specifications, and knitted structures is still required to further establish broader structure–property relationships. Since the numerical simulation provides deterministic outputs rather than independent experimental populations, conventional statistical significance tests (e.g., t-test or ANOVA) between simulation and experiment are not directly applicable. Therefore, the statistical analysis in this study focuses on experimental repeatability and uncertainty quantification through repeated measurements, standard deviation, and confidence interval analysis.

## 4. Conclusions

This study proposes a rule-driven three-dimensional geometric modeling framework for polymer-fiber weft-knitted structures oriented toward numerical simulation. The proposed method transforms knitting actions into parametric yarn-centerline representations and provides an automated approach for generating continuous and meshable textile geometries. The main conclusions are summarized as follows:

(1) A unified geometric representation based on knitting-action rules and parametric yarn centerlines was established. The proposed framework enables automated generation of various single-bed and double-bed knitted structures, reducing the dependence on manually adjusted control points and structure-specific modeling procedures.
(2) The proposed method demonstrates good geometric extensibility and simulation compatibility. Multiple knitted architectures, including plain, tuck, plated, rib, interlock, cardigan, and purl structures, can be represented within the same modeling framework while maintaining continuous yarn paths and reliable mesh generation.
(3) The feasibility of applying the generated knitted geometries to moisture-transfer simulation was verified through comparison between numerical predictions and experimental measurements. The results demonstrate that the proposed geometry-generation strategy can provide reliable simulation-ready models for structure–property analysis of polymer-fiber knitted textiles.

Overall, the proposed method provides a unified, structurally extensible, and simulation-preprocessing-compatible three-dimensional modeling approach for complex polymer-fiber weft-knitted structures. It should be noted that the present model is primarily a geometry-driven framework, and the current experimental validation focuses on one representative polymer-fiber knitted fabric to demonstrate the feasibility of the proposed approach rather than to establish a comprehensive material-performance database. The proposed geometric generation strategy is independent of specific polymer compositions and yarn materials, and material-related parameters can be flexibly updated in subsequent multiphysics simulations. However, the current framework does not explicitly consider mechanical factors such as yarn tension, contact friction, cross-sectional compression, relaxation recovery, or polymer-specific effects including swelling, surface ageing, and material-dependent wetting variation. Future work will further improve the predictive capability by incorporating measured loop morphology, yarn-level polymer material properties, and mechanical constraints, and will extend the validation to different polymer fibers, yarn structures, and experimental datasets to establish more comprehensive structure–property relationships for diverse polymeric textile systems.

## Figures and Tables

**Table 1:** Comparison of recent studies on polymer composites and knitted textile modeling approaches, highlighting the research gap addressed in this work.

Research Category	Main Objective	Typical Approaches	Main Achievements	Remaining Gap
Hybrid polymer/natural fiber composites	Improve material properties through reinforcement design	Fiber hybridization, interface modification, composite fabrication [12,13]	Enhanced mechanical and functional performance	Limited consideration of complex textile geometry
Fiber-reinforced polymer simulation	Predict composite mechanical behavior	FEM, homogenization, multiscale simulation [14,15]	Established structure–property relationships	Reinforcement geometry usually predefined
Classical knitted fabric modeling	Represent loop morphology	Peirce model and analytical loop models [16,17,18,19]	Effective description of basic knitted structures	Difficult to extend to complex knitting actions
Curve-based yarn modeling	Improve geometric flexibility	Piecewise curves, spline, NURBS methods [5,20,21,22,23,24,25,26,27]	Smooth yarn-path reconstruction	Dependence on manual control points and limited automation
Multiscale knitted textile simulation	Predict mechanical and functional performance	Yarn/fabric coupled simulation [28]	Enables numerical analysis of knitted materials	Requires reliable meshable geometric input
Present work	Simulation-oriented digital representation of polymer-fiber knitted structures	Rule-driven action encoding + parametric centerline modeling	Automated generation of complex knitted geometries with improved continuity and meshability	Further coupling with yarn mechanics and material-specific effects is required

**Table 2:** Nomenclature of symbols and abbreviations used in this study.

Symbol	Definition	Unit
*a*	Horizontal amplitude of yarn loop centerline	mm
*h*	Vertical loop height	mm
*d*	Yarn thickness-related amplitude parameter	mm
*r*	Equivalent yarn radius	mm
*t*	Parametric variable for centerline generation	/
*x*,*y*,*z*	Three-dimensional coordinates of yarn centerline	mm
*P*	Knitting pattern matrix describing stitch actions	/
*N_r_*	Number of pattern rows	/
*N_c_*	Number of pattern columns	/
*i*,*j*	Row and column indices in pattern matrix	/
*L*	Yarn centerline length	mm
*Q_min_*	Minimum element quality of finite element mesh	/
*Q_avg_*	Average element quality of finite element mesh	/
*E_L_*	Relative error of yarn length	%
*E_P_*	Key-point position error	%
*C* ^1^	First-order geometric continuity	/
*O_F_*	One-way liquid transport capability from face side	/
*O_S_*	One-way liquid transport capability from back side	/
*ΔO*	Difference between face-side and back-side transport capability	/
SD	Standard deviation	/
CI	Confidence interval	/
RVE	Representative volume element	/
COMSOL	Multiphysics simulation software platform	/
AATCC	American Association of Textile Chemists and Colorists	/

**Table 3:** Comparison of centerline geometric stability and geometric differences between different modeling methods.

Structure Type	Modeling Method	Number of Centerline Points	Minimum Path Distance/mm	Curvature-Jump Rate/%	Maximum Bending Angle/°	Mean Key-Point Error/%
Plain stitch	Interpolated-curve method	22	0.2246	40.78	82.53	/
	Proposed method	128	0.1244	0.00	10.39	0.3449
Fully plated plain stitch	Interpolated-curve method	60	0.2602	61.54	61.04	/
	Proposed method	128	0.1478	0.00	14.21	0.0131
Single-needle double-course tuck	Interpolated-curve method	18	0.2176	56.25	112.37	/
	Proposed method	642	0.0151	0.31	51.07	1.1084
Double-face tuck	Interpolated-curve method	45	0.0627	69.23	111.33	/
	Proposed method	773	0.0366	0.26	47.09	0.9103

**Table 4:** Statistical analysis of experimental moisture-transfer measurements and comparison with simulation results.

Direction	Experimental Mean	SD	95% CI	Simulation Result	Relative Error
Face side	−120.2257	1.0159	[−122.7493, −117.7022]	−122.3187	1.74%
Back side	122.8615	0.8855	[120.6618, 125.0612]	122.2472	0.50%

## Data Availability

The original contributions presented in this study are included in the article. Further inquiries can be directed to the corresponding author.
